# Developmental and epileptic encephalopathy related to a heterozygous variant of the *RHOBTB2* gene: A case report from French Guiana

**DOI:** 10.1002/mgg3.1929

**Published:** 2022-03-21

**Authors:** Antoine Defo, Alain Verloes, Narcisse Elenga

**Affiliations:** ^1^ Pediatric Medicine and Surgery Centre Hospitalier de Cayenne Cayenne Cedex French Guiana; ^2^ Department of Clinical Genetics Hôpital Robert Debré Paris France

## Abstract

Here we report a case of developmental and epileptic encephalopathy related to RHOBTB2 gene mutation in a ten‐month old infant in French Guiana. Although the 28 previously reported cases had early‐onset epilepsy and severe intellectual disability, here the reported individual presented with late postnatal onset of microcephaly and the absence of cortical atrophy on MRI. The publication of cases of such a rare form of developmental and epileptic encephalopathy will eventually allow us to better understand the mechanism by which RHOBTB2 misregulation could induce severe and atypical neurological disorders.

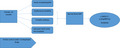

To the editor,

Accurate diagnosis of the type of epilepsy is crucial for effective treatment. Genetic mutations are thought to play key roles in the development of certain epilepsies. Next‐generation sequencing technologies have greatly facilitated the identification and confirmation of disease‐related genes. Neurodevelopmental disorders are mediated by several pathophysiological mechanisms, including disturbances in cytoskeleton plasticity and synaptic plasticity due to Ca^2+^ and Ras homolog (Rho) GTPase‐mediated signaling. The *RHOBTB2* gene encodes Rho‐related BTB domain‐containing protein 2, which is an atypical Rho GTPase that is a substrate of the Cullin‐3 (CUL3)‐based ubiquitin ligase complex. Atypical Rho GTPases (such as RHOBTB2) may play a role in neurological function and dendrite development. Precise regulation of RHOBTB2 levels is essential for normal brain function. Differential diagnoses are complicated as there are multiple etiologies of epileptic and neurodevelopmental encephalopathy, which involve genetic, structural cerebral, neurometabolic, as well as still unknown factors. In terms of genetic etiologies, it is always relevant to the search for variants of the *RHOBTB2* gene. Since the initial findings of a de novo missense variant in *RHOBTB2* in individuals with early‐onset epilepsy and severe intellectual disability (Straub et al., 2018), there have been few reported cases.

Here, we report a case of developmental and epileptic encephalopathy related to *RHOBTB2* gene mutation in a 10‐month‐old infant in French Guiana. Our patient is currently a 10‐month‐old female with no personal or family history, referred to us at 3 months of age as a result of serial tonic–clonic epileptic seizures with complex and varied semiology. We noted early occurrence of epileptic activity in our patient (with approximately 4–5 seizures/month despite having started an antiepileptic tritherapy with the optimal posologies) of varied semiology: generalized tonic–clonic seizures with labial cyanosis as well as brief oculogyric seizures sometimes followed by postictal asthenia and focal clonic seizures of the right upper limb. She was born after a full‐term pregnancy to non‐consanguineous parents. Her neurological development was notable with microcephaly at −2.5 SD observed from 9 months of age (head circumference was normal at birth) and moderate psychomotor retardation (absence of sitting without support at 10 months, no canonical babbling, and social contact below age), axial hypotonia, and selective eating disorder (severe anorexia). The deep tendon reflexes were normal and symmetrical, and the Babinski reflex was negative. Her global development quotient (Brunet‐Lézine Early Childhood Psychomotor Development Scale) gradually deteriorated and was comparable to that of a 6‐month‐old infant. Several EEGs were performed, showing little slowed electrogenesis for age (delta rhythm) and diffuse polyspikes on the right hemisphere at 3 and 10 months (Figure 1). MRI brain imaging was normal. The ophthalmic exam and funduscopy were normal. A complete blood count revealed an RBC of 2.03 × 10^12^/L, a WBC of 17.70 × 10^9^/L, and a platelet count of 294 × 10^9^/L, with normal hemostasis tests. The liver enzyme levels were normal, as were the total bilirubin, serum creatinine, and metabolic balance (plasma amino acid chromatography, plasma acylcarnitine profile, plasma total, and free carnitine, lactates in plasma and cerebrospinal fluid). Next‐generation sequencing for diagnosis by screening of a panel of epilepsy genes, carried out at the genetic laboratory of Hopital La Timone in Marseille, revealed the presence of a de novo variant in exon 5 of *RHOBTB2* (NM_015178.3): c.1465C>T: p.(Arg489Trp):Hg19 chr8:22865223C>T. Thus, our patient has a missense variant that affects arginine 489, located between the two BTB domains. Interestingly, another substitution of the same amino acid (p.Arg489Gly), rated class 4 (likely pathogenic) according to the ACMG guidelines, has been reported in two unpublished patients.(Clinvar, n.d.) The pathogenicity of the variant was established by biocomputational analysis using the following software: Torrent Suite (Thermo Fisher), VarAFT: ANNOVAR ± UMD‐Predictor annotation. This epileptic encephalopathy has been shown to be drug resistant after multiple failures of mono‐and then bitherapy antiepileptics as well as antiepileptic vitamin therapy. Currently, she is under a triple antiepileptic medication with partial control of her epileptic seizures. Her clinical features are detailed in Table 1.

**FIGURE 1:**
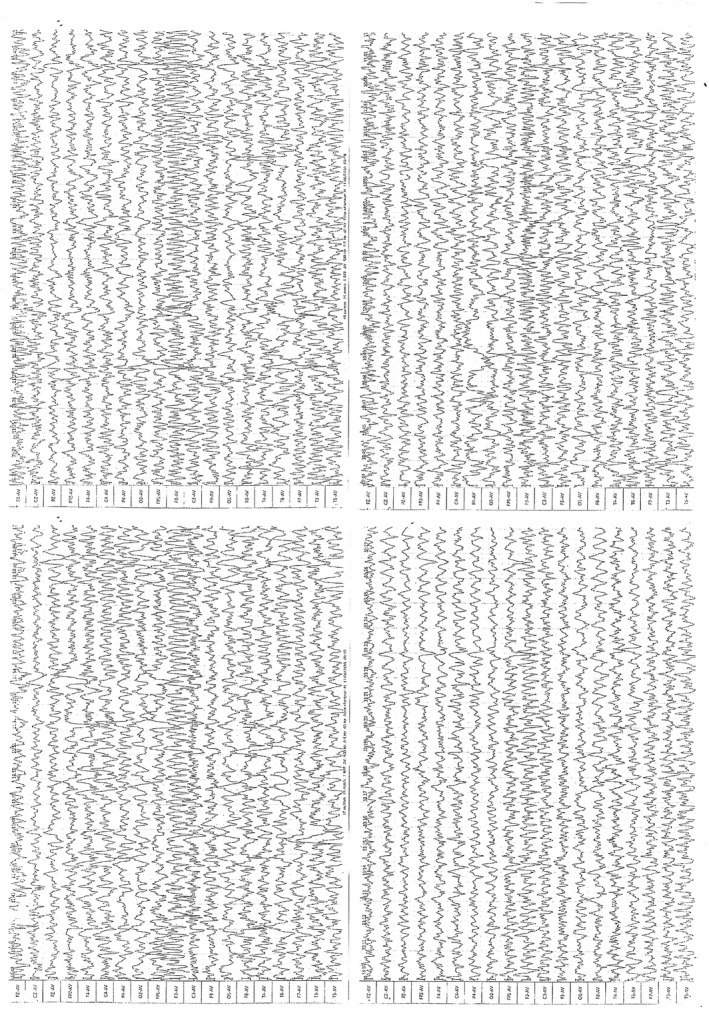
EEG showing diffuse polyspikes on the right hemisphere

**TABLE 1:** Clinical features of individual with de novo *RHOBTB2* variant

Sex, age	Female, 10 months
Variant	c.1465C>T: p.(Arg489Trp)
Gestation	40 weeks
Weight	3330 g (mean) and 8.070 kg (−2 SD) at 10 months
Head circumference	34.5 cm (−1 SD) at birth and 41 cm (−2.5 SD) at 10 months
Seizure onset	3 months
Seizure type	Clonic movements and cyanosis (focal and generalized) serial seizures, status epilepticus
Acute encephalopathy	Prolonged altered consciousness and permanently abnormal movements since 3 months
Seizure control	Weekly; partially controlled with topiramate, Valproic acid and Oxcarbazepine
EEG	Diffuse polyspikes on right hemisphere at 3 and 10 months
Funduscopy	Normal
Brain MRI	Normal at 3 months and 10 months
Intellectual disability	Moderate
Motor function	Head control at 7 months; sitting at 12 months
Neurological examination	Poor coordination
Movement disorders	Athetoid movement

Abbreviations: EEG, electroencephalogram; MRI, magnetic resonance imaging.

De novo missense variants in *RHOBTB2* were recently reported (Clinvar, n.d.; Belal et al., 2018; Knijnenburg et al., 2020; Straub et al., 2018; Zagaglia et al., 2021) in 28 individuals with a phenotype including epilepsy, moderate to severe intellectual disability that was sometimes associated with developmental regression or plateauing, postnatal microcephaly, and movement disorders. All of these variants cluster in or close to either the first or second BTB domain, at positions important for stabilizing interactions within the domain or for dimer formation as well as impaired degradation of RHOBTB2 by the proteasome. The severity of the seizure types varied, from early‐onset epilepsy to focal, complex partial, and generalized tonic–clonic seizures. Some patients had only late‐onset febrile seizures. At least five patients were reported to have acute encephalopathy and epileptic seizures triggered by hyperthermia or head trauma (Belal et al., 2018; Knijnenburg et al., 2020). The movement disorder appears characteristic, associating ataxia, dystonia and paroxysmal chorea‐like movements, and alternating hemiplegia of childhood (AHC). The AHC was delayed compared with the phenotype of *ATP1A3*‐related AHC (Zagaglia et al., 2021). One of the unpublished patients has microcephaly, intellectual disability, seizures, optic atrophy, and gait ataxia.

In conclusion, we have identified a de novo variant of *RHOBTB2* as a rare genetic cause of epileptic encephalopathy. Our case report clearly supports the conclusions of previous studies (Clinvar, n.d.; Belal et al., 2018; Knijnenburg et al., 2020; Niu et al., 2021; Straub et al., 2018; Zagaglia et al., 2021), albeit with some specificities. However, how *RHOBTB2* variants lead to epileptic encephalopathy remains unknown. The mechanism by which *RHOBTB2* misregulation induces severe and atypical neurological disorders needs to be further explored.

## CONFLICT OF INTEREST

The authors have no conflicts of interest to report.

## ETHICS STATEMENT

The procedures used in this study were reviewed and approved by the Ethics Committee of the Cayenne Hospital.

## PARENT CONSENT

Written informed consent for publication was provided by the patient's parents.

## Data Availability

The data supporting the findings of this study are available from the corresponding author upon reasonable request.
